# Low-Density Materials for Particle Accelerator Applications: Challenges, Capabilities, and Future Directions

**DOI:** 10.3390/ma19184008

**Published:** 2026-09-20

**Authors:** Alexander J. G. Lunt, Elisabeth Sena-Welker, Chiara Pasquino, Philippe Poulin, Mariusz Sapinski, Stefano Sgobba, Thierry Stora, Raymond Veness

**Affiliations:** 1 Department of Mechanical Engineering, University of Bath, Bath BA2 7AY, UK; 2 CERN, European Organization for Nuclear Research, Esplanade des Particules 1, 1217 Meyrin, Switzerland; elisabeth-sena.welker@cern.ch (E.S.-W.); chiara.pasquino@cern.ch (C.P.); stefano.sgobba@cern.ch (S.S.);; 3 Centre de Recherche Paul Pascal, University Bordeaux, CNRS, UMR 5031, F-33600 Pessac, France; 4 Paul Scherrer Institute (PSI), 5232 Villigen, Switzerland

**Keywords:** particle accelerators, carbon nanotubes, beam instrumentation, beam–matter interactions, multiscale modelling, nanostructured materials, materials characterisation

## Abstract

**Highlights:**

- Low-density materials offer significant potential for accelerator systems.
- Multiscale structures strongly influence material behaviour.
- Community priorities for future research were identified.
- Reproducibility and validation remain major challenges.
- Future accelerators will drive demand for next-generation instrumentation.
- Improved material qualification methods are needed.
- Better modelling can support material development and deployment.

**Abstract:**

Low-density materials are emerging as a critical enabling technology for next-generation particle accelerator systems, offering unique combinations of low areal density, high thermal resilience, and tuneable functional properties. To address the challenges associated with their development and deployment, an international, multidisciplinary community spanning accelerator physics, materials science, and engineering convened to assess the current state of the field and define future research directions. The discussions highlighted significant progress in the development of carbon-based materials, including carbon nanotube fibres, graphene, and related nanostructured systems, alongside advances in fabrication, characterisation, and modelling capabilities. However, key challenges remain in achieving reproducible material quality, controlling impurities and microstructure, and bridging the gap between laboratory-scale demonstrations and reliable operation in accelerator environments. Limitations in predictive modelling and the availability of validated material data were also identified as critical barriers. The workshop underscored the importance of coordinated efforts in standardisation, testing under realistic conditions, and integration of experimental and simulation approaches. Emerging opportunities were identified in advanced beam instrumentation, high-performance targets, and novel functional materials. Overall, the outcomes emphasise the need for sustained collaboration and strategic investment to realise the full potential of low-density materials in future accelerator applications.

## 1. Workshop Overview

The Low-Density Materials for Particle Accelerator Applications workshop brought together an international and interdisciplinary community of researchers spanning accelerator physics, materials science, chemistry, and engineering. The programme was designed to foster dialogue between material developers, experimentalists, and end-users, with a particular focus on bridging the gap between emerging material technologies and their deployment in operational accelerator environments.

Contributions ranged from application-driven studies in beam instrumentation and targets to advances in synthesis, processing, characterisation, and modelling of low-density materials. A central aim of the workshop was to provide a coherent framework linking application requirements to material capabilities, while identifying the key technical barriers limiting deployment. The discussions emphasised the importance of integrating experimental insight with modelling approaches and highlighted the need for coordinated community efforts to address challenges related to reproducibility, standardisation, and validation.

An overview of the scientific contributions and the participants of the workshop is provided in the Supplementary Material as well as Table A1 and Table A2 of Appendix A, respectively.

### 1.1. Background and Motivation

The next generation of accelerators, such as the High-Luminosity LHC (HL-LHC) Project or the proposed Future Circular Collider (FCC), will operate with beams that are significantly higher in energy than anything used today, pushing the current technologies to the limit. HL-LHC, for example, aims to increase its instantaneous luminosity (rate of collisions) by a factor of five, creating much harsher beam–material interaction conditions [1]. Looking further ahead, the FCC-hh project allows for proton–proton collisions at energies up to 100 TeV, exploring new physics and discoveries while simultaneously imposing extreme demands on the accelerator components [2].

As the beam energy increases, conventional materials are approaching their operational limits due to increasing beam intensities and reduced beam sizes. This has driven the exploration of alternative materials with improved thermal resilience and reduced perturbation of the beam.

Low-density materials, particularly carbon-based systems such as carbon nanotube (CNT) fibers, graphene, and related nanostructured materials, are increasingly seen as enabling technologies for advanced accelerator applications. Their low areal density reduces beam interaction, while their thermal, mechanical, and functional properties offer potential advantages over conventional materials in high-power and high-radiation environments [3].

**Figure 1:**
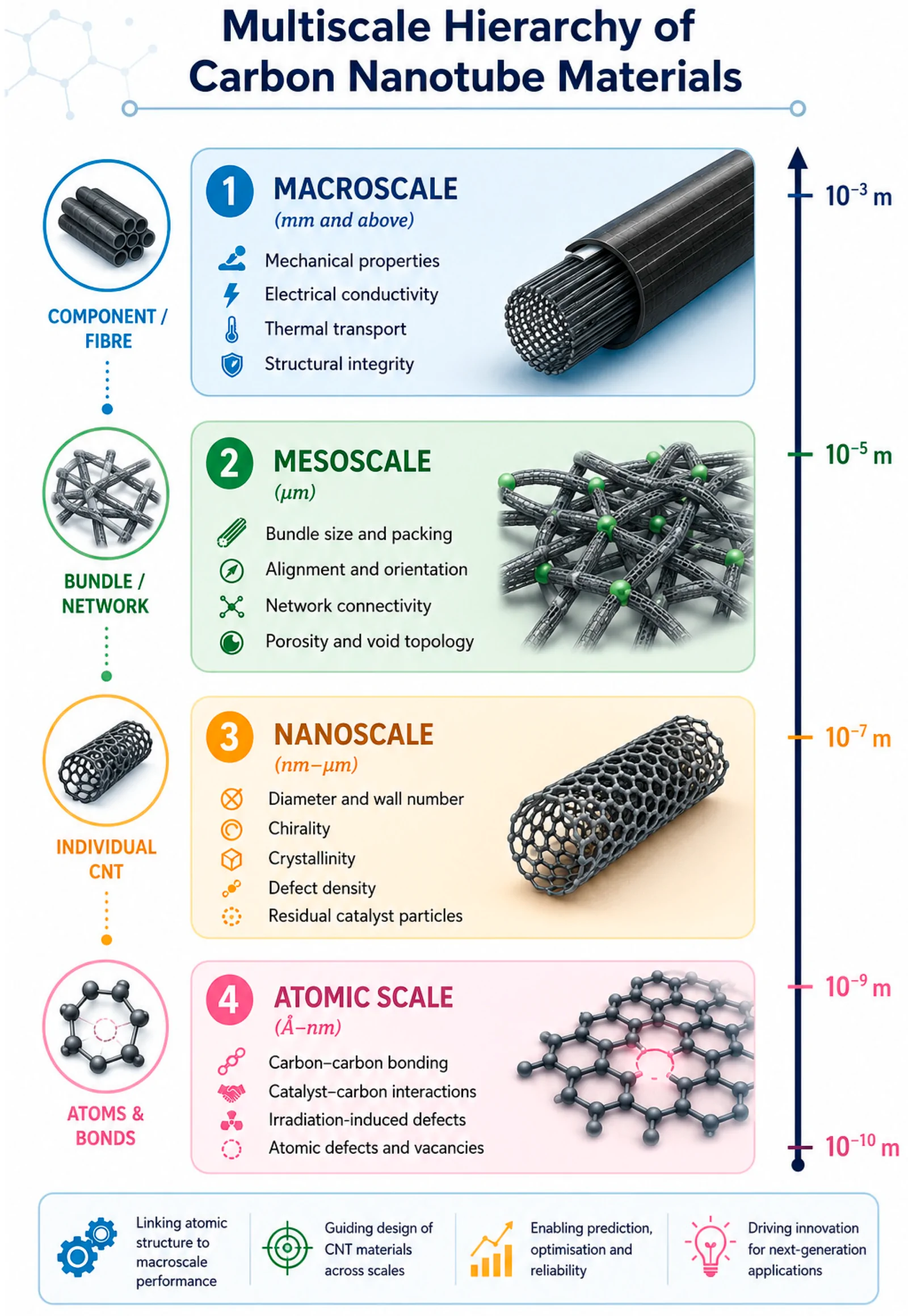
Example of hierarchical structure of a low-dimensional material (CNTs) across atomic, nanoscale, mesoscale, and macroscale length scales. Adapted from workshop presentation material provided by J.A. Elliott at the Low-Density Materials Workshop 2026 and from concepts described in [8,9]. The graphical layout and certain visual elements were developed with the assistance of Microsoft 365 Copilot and subsequently reviewed and edited by the authors.

However, it is increasingly evident that the performance of these materials is not governed solely by their intrinsic properties at the atomic scale. Instead, their effective performance arises from the structural features across multiple length scales. An example is the hierarchical structure of CNT fibers (Figure 1): although individual single-walled CNTs exhibit exceptional intrinsic properties, macroscopic wires are composed of many interacting CNTs arranged in bundles and layers, which significantly modifies their overall behaviour [4]. As a result, the realised performance can differ substantially from the ideal intrinsic properties.

It is therefore essential to understand these effects for the reliable implementation of nanostructured materials in future high-energy accelerators, where material response under extreme conditions is strongly influenced by microstructure and impurities. These challenges arise across different beam-intercepting devices, targets, and thin-film systems, and introduce a significant uncertainty in predicting behaviour, particularly under irradiation and thermal cycling [5,6,7].

From a production perspective, advances in synthesis techniques, including floating catalyst chemical vapour deposition and fibre spinning, have enabled increasing control over material structure. Nevertheless, achieving consistent and reproducible materials at scale remains a major limitation, with strong sensitivity to processing conditions and residual catalyst content [10].

In parallel, developments in experimental characterization have provided new insights into structure–property relationships, with techniques capable of probing materials across multiple length scales and under realistic conditions [11]. However, the lack of standardised methodologies and comparable datasets limits the ability to validate models and translate results across different studies.

Finally, while modelling and simulation approaches, ranging from atomistic methods to continuum-scale descriptions, are becoming increasingly sophisticated, their predictive capability is often constrained by insufficient input data and limited experimental validation [8]. This highlights the need for tighter coupling between experimental and computational efforts.

Taken together, these challenges motivate a coordinated and multidisciplinary approach to the development of low-density materials, with an emphasis on reproducibility, validation, and integration into real-world accelerator systems.

### 1.2. Objectives

The workshop was conceived to provide a focused and application-driven assessment of the role of low-density materials in current and future particle accelerator systems. Bringing together expertise across multiple disciplines, it aimed to move beyond isolated developments in materials or instrumentation and instead establish a coherent understanding of how emerging material systems can be effectively translated into operational environments. Central to this effort was the need to connect application requirements with material capabilities, while identifying the key limitations that currently prevent wider adoption. To achieve this, the workshop focused on a small number of core objectives:

- **Assess the current state of the field**, with particular emphasis on applications in beam instrumentation, targets, and related accelerator components.
- **Identify the principal technical challenges,** including limitations in performance, reproducibility, and reliability under extreme conditions.
- **Evaluate gaps in capability**, spanning material production, characterisation techniques, and predictive modelling approaches.
- **Improve understanding of structure–property relationships,** particularly the role of micro- and mesoscale features in determining material behaviour.
- **Establish priorities for future research and collaboration,** including opportunities for coordinated development and shared approaches.

Collectively, these objectives were intended to provide a foundation for a more integrated and strategic approach to low-density materials, supporting both near-term applications and longer-term innovation. The outcomes of these discussions serve to highlight not only the potential of these materials, but also the need for closer alignment across the community to realise that potential in practice.

### 1.3. Format and Participation

The workshop was held over two days at the University of Bath and brought together an international group of participants from academia, research institutes, and industry. The programme was structured around a series of themed sessions, combining invited presentations with extended discussion periods to encourage interaction across disciplines and between material developers and end-users.

Sessions were organised to reflect the key stages in the development and deployment of low-density materials, progressing from applications and requirements, through production and processing capabilities, to characterisation, modelling, and future strategic directions. Each session was chaired by a member of the steering committee to ensure continuity and to facilitate focused discussion.

The workshop included contributions from a broad range of institutions, including major accelerator laboratories, universities, and specialised materials research centres. The participant group combined expertise in accelerator physics, material synthesis, nanotechnology, characterisation, and simulation, enabling a comprehensive and multidisciplinary perspective on the challenges addressed.

The format placed particular emphasis on discussion and exchange, with dedicated time allocated for questions, informal interactions, and follow-up engagement. This structure was intended to maximise cross-fertilisation between different areas of expertise and to support the development of future collaborations within the community.

## 2. Applications: State of the Art and Community Needs

Low-density materials are increasingly central to the development of particle accelerator technologies, where performance requirements are driven by extreme beam conditions, high power densities, and the need for minimal beam perturbation. Across a range of systems, including beam diagnostics, beam-intercepting devices, and emerging functional components, these materials offer unique advantages due to their low areal density and tuneable properties. At the same time, their deployment introduces new challenges related to material control, reproducibility, and integration. This section provides an overview of current and emerging applications, the state of the art in material capability, and the key challenges and needs identified through the workshop discussions.

### 2.1. Current Application Landscape

Low-density materials are currently deployed, or under active development, across a wide range of particle accelerator systems, where they enable operation under increasingly demanding beam conditions. Their importance arises from the need to minimise beam interaction while maintaining adequate mechanical, thermal, and functional performance, particularly in environments characterised by high power density, rapid cycling, and irradiation. As accelerator facilities continue to push towards higher intensities and smaller beam sizes, the role of materials has shifted from a secondary design consideration to a primary performance-limiting factor, driving the exploration of alternative material systems with reduced density and tailored properties [12].

One of the most established application areas is beam diagnostics, where instruments are categorized either as interceptive (physically placing material directly into the particle beam) or non-interceptive (passively monitoring the beam via emitted synchrotron radiation or electromagnetic fields). These systems rely fundamentally on beam–matter interaction to extract spatial or temporal information, placing strict constraints on material thickness, density, and stability. A classic example of an interceptive device is the wire scanner, which sweeps a solid wire across the particle beam, generating particle showers proportional to the beam’s transversal profile.

**Figure 2:**
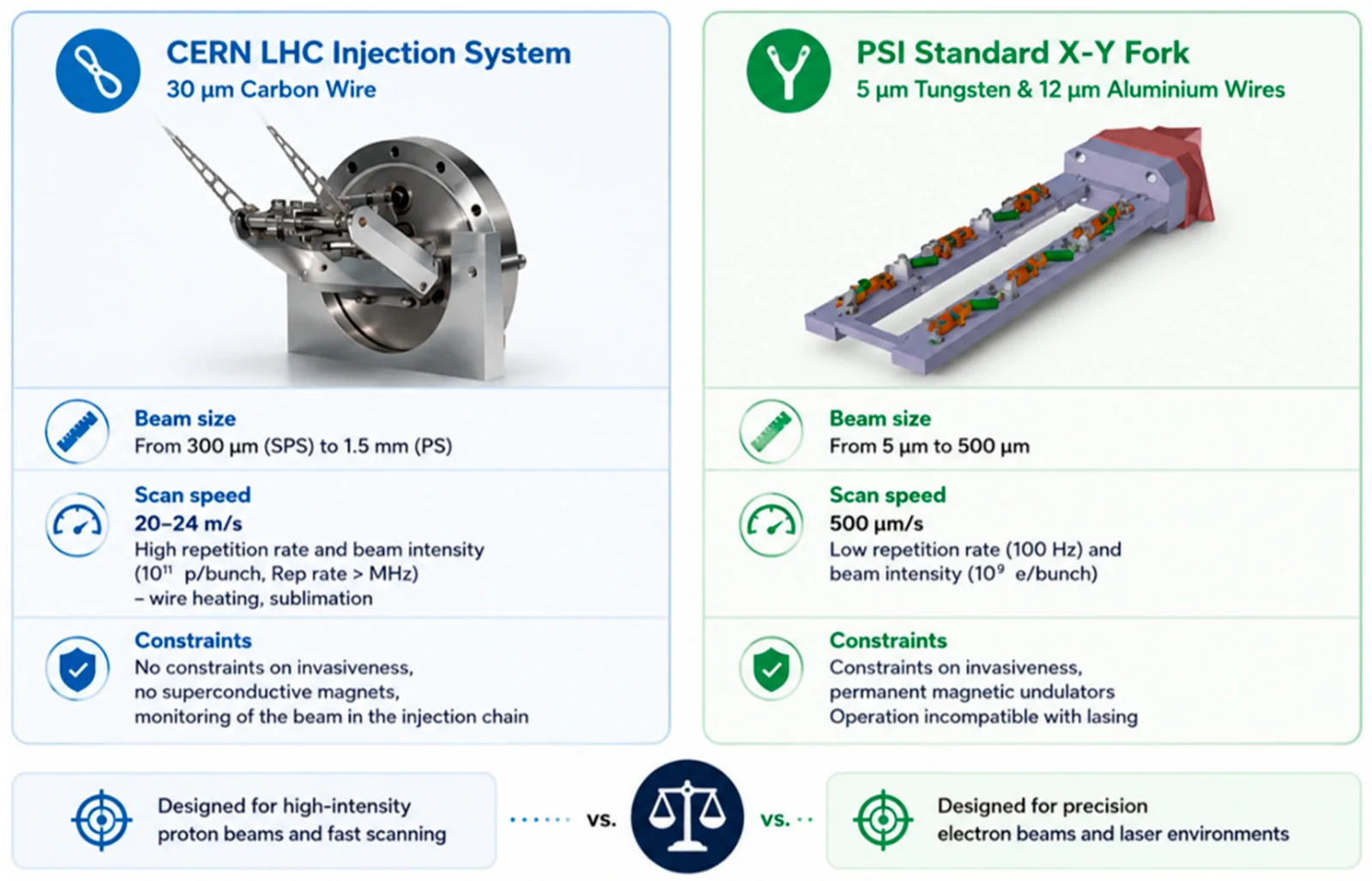
Examples of low-density material applications in wire scanner applications. Adapted from F.M. Addesa, workshop presentation, Low-Density Materials Workshop 2026.

Conventionally, metallic wires or carbon fibres are used as sensing elements, but their performance is increasingly limited by thermal damage, sublimation, and mechanical fatigue under repeated beam exposure (Figure 2). As beam sizes approach the micrometre scale, even small perturbations due to scattering or energy deposition become significant, necessitating the use of thinner and lower-density materials to preserve measurement fidelity [13,14]. This has driven a strong shift towards nanostructured materials and ultra-fine sensing elements.

Low-density materials are also critical in beam-intercepting devices, including imaging screens, stripping foils, and collimation systems. In these applications, materials must withstand direct interaction with high-intensity beams while maintaining stable response characteristics. For example, stripping foils used in high-power proton accelerators must be sufficiently thin to achieve efficient charge exchange, while also resisting rapid degradation under thermal and radiation loading. Similarly, beam imaging screens must balance light output, resolution, and survivability, often under conditions where localised heating and damage are unavoidable. Reducing material density offers clear advantages in lowering energy deposition, but this is often accompanied by reduced mechanical robustness and increased sensitivity to defects or structural heterogeneity [15].

Another application is in facilities like CERN-ISOLDE, where proton beams collide with specialised targets to create rare radioactive isotopes for fundamental physics and medical research. Low-density and porous materials are increasingly being explored to manage extreme thermal loads and mitigate stress accumulation of the target systems.

These systems operate under some of the most demanding conditions in accelerator environments, with sustained energy deposition leading to significant temperature gradients and structural evolution [16]. Here, low-density materials may provide improved thermal shock resistance and reduced peak stress, but their long-term behaviour remains difficult to predict, particularly in the presence of irradiation-induced changes and microstructural instability.

Beyond these established applications, there is growing interest in thin-film and two-dimensional material systems, including graphene-based materials and related nanostructures. These materials offer the potential for extremely low interaction thickness combined with highly tuneable electronic, optical, and thermal properties. As such, they are increasingly considered for next-generation diagnostic systems, photon interaction platforms, and sensing applications, where their reduced dimensionality enables new modes of operation. However, integrating such materials into mechanically robust and scalable systems remains a significant challenge, particularly when considering handling, mounting, and environmental stability [17,18].

A further emerging direction is the use of nanostructured and hierarchical materials, such as carbon nanotube fibres and arrays, which combine low density with high aspect ratio and unique functional properties. These materials are being explored not only as replacements for conventional components, but also as enabling technologies for new accelerator concepts. For example, their use as field emission cathodes highlights a shift towards materials that actively participate in beam generation and manipulation, rather than serving solely as passive elements [19,20].

Across these diverse application areas, a unifying theme is the increasing importance of material architecture at multiple length scales. In many low-density systems, properties are not determined solely by composition, but by hierarchical structure, including alignment, porosity, and defect distribution. This introduces both significant opportunities for tailoring performance and substantial challenges in achieving reproducibility and control. As a result, the current application landscape is characterised not only by expanding opportunities, but also by increasing complexity in understanding and predicting material behaviour under operational conditions.

### 2.2. Key Technical Challenges

Despite the significant progress in the development of low-density materials for accelerator applications, several fundamental technical challenges continue to limit their widespread deployment. These challenges are not confined to a single application, but instead arise consistently across diagnostics, beam-intercepting systems, and emerging material concepts, reflecting the complex interaction between material structure, processing, and operational environment.

A central issue is the strong dependence of material performance on microstructure and processing history. In many low-density systems, particularly those based on carbon nanotubes and related nanostructures, properties such as thermal conductivity, mechanical strength, and electrical behaviour are governed by alignment, packing density, porosity, and the presence of defects. Even small variations in synthesis conditions can lead to significant differences in performance, making it difficult to ensure consistency between samples or across production batches [7,18,21]. This variability undermines confidence in both experimental results and the reliability of deployed components.

Closely linked to this is the challenge of impurities and contamination. Residual metal catalyst particles, amorphous carbon, and structural inhomogeneities can dominate the behaviour of CNT-based systems, particularly under beam interaction, where localised heating effects can be exacerbated. These defects may significantly alter thermal transport pathways and accelerate material degradation, making their control essential for reliability.

**Figure 3:**
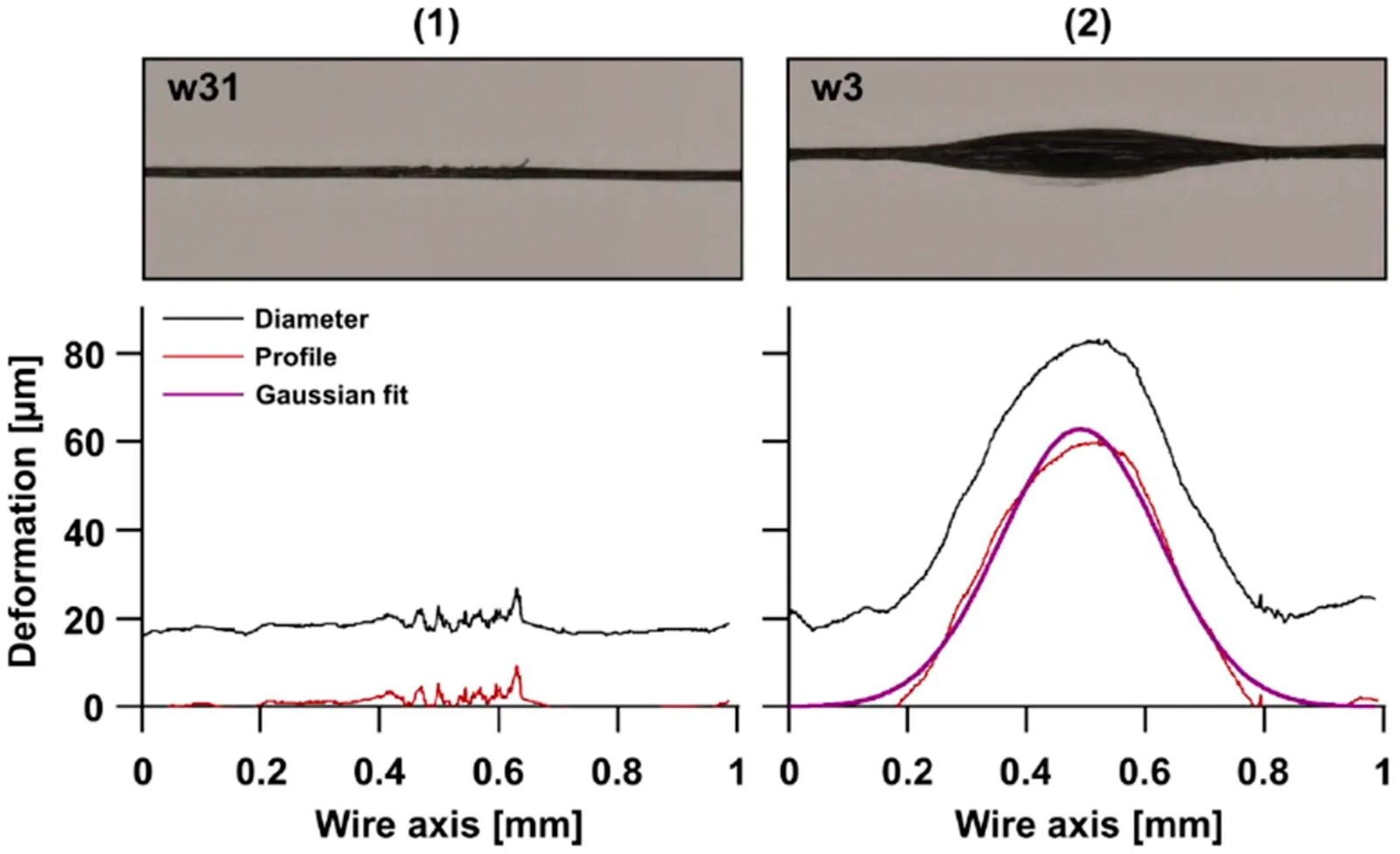
Post-irradiation deformation of CNT wires [22]. (**1**) Irradiated wire showing limited changes to cross section; (**2**) Irradiated wire showing increase in cross section.

A further challenge arises from the extreme operational environments encountered in accelerator systems. Materials are routinely subjected to high power densities, rapid thermal cycling, and intense radiation fields, often simultaneously. Under such conditions, thermal transport is strongly influenced by anisotropy and defect structures, while repeated beam exposure can lead to cumulative effects such as sublimation, embrittlement, or structural collapse (Figure 3). The interaction between these processes remains difficult to predict, particularly in hierarchical or porous systems where multiple length scales are involved in the operation [3,22].

Another critical limitation is the lack of reliable and transferable material data to support modelling and design. While computational approaches are increasingly used to simulate beam–material interaction and optimise component performance, their predictive capability depends heavily on the availability of accurate input parameters [23]. In many cases, material properties are derived from small-scale or idealised samples, which are not representative of real systems. This mismatch limits the ability to validate models and reduces confidence in simulation-driven design decisions.

Scaling from laboratory demonstration to operational implementation presents an additional challenge. Materials that exhibit promising properties under controlled conditions often prove difficult to reproduce consistently at larger scales. Variability in synthesis, handling, and integration can introduce defects or inconsistencies that significantly affect performance. This is particularly critical in applications such as wire scanners or thin-film systems, where small deviations in geometry or material properties can have a disproportionate impact on behaviour [3,18].

Finally, integration into existing accelerator systems introduces a further layer of complexity. New materials must be compatible not only with mechanical and thermal requirements, but also with vacuum conditions, alignment tolerances, and operational constraints. In many cases, adopting a new material requires changes to system design or operational procedures, which can limit implementation despite clear performance advantages [13].

Taken together, these challenges highlight that the limitations of low-density materials arise not only from intrinsic properties, but from the interplay between structure, processing, and environment. Addressing these issues will require coordinated advances across materials development, experimental validation, and system-level integration, forming the basis for the community needs outlined in the following section.

### 2.3. Community Needs

The technical challenges identified across applications point to a clear set of shared needs within the community, reflecting the fact that many of the current limitations are not isolated to specific materials or devices, but arise from systemic gaps in capability, coordination, and methodology. Addressing these issues will require a more integrated approach that links materials development, experimental validation, and accelerator implementation.

A primary need is the establishment of improved reproducibility and standardisation in both material production and characterisation. As discussed in Section 3.3 the performance of low-density materials is highly sensitive to processing conditions, with properties often varying significantly between nominally similar samples. Without consistent protocols for synthesis, handling, and testing, it remains difficult to compare results across different groups or to benchmark materials reliably. This lack of standardisation limits both scientific progress and the confidence required for deployment in operational systems, and highlights the importance of developing agreed methodologies and reference materials [24].

Closely related to this is the need for more comprehensive and transferable material datasets. While a large body of experimental data exists, it is often fragmented, incomplete, or not directly applicable to modelling and design. There is a gap between the quantities that can be measured experimentally and those required as inputs for simulation tools. Bridging this gap will require coordinated efforts to define relevant material parameters, collect high-quality datasets, and ensure traceability to processing history and experimental conditions [25,26]. Such efforts are essential to enable predictive modelling and to support the design of accelerator components incorporating these materials.

Another critical area is the development of testing methodologies that accurately reflect operational conditions. Many current studies rely on laboratory-scale experiments that do not fully capture the complexity of real accelerator environments, including time structure, cumulative beam exposure, and coupled thermal and radiation effects. The community would benefit from expanded use of dedicated test facilities, as well as the development of standardised test protocols that allow meaningful comparison between different materials and studies. This includes both high-intensity beam testing and complementary approaches such as in situ characterisation and multi-physics experimentation.

There is also a clear need for stronger integration between experimental and modelling activities. At present, these efforts are often carried out in parallel, with limited feedback between them. Improving this integration would allow experimental results to directly inform model development, while modelling could guide experimental design and parameter selection. Such an approach would accelerate the development cycle and improve confidence in both material behaviour and system-level performance, particularly for complex or hierarchical materials where intuition alone is insufficient.

Beyond technical aspects, the workshop highlighted the importance of increased coordination and collaboration across the community. The challenges associated with low-density materials are inherently multidisciplinary, requiring expertise spanning materials science, accelerator physics, chemistry, and engineering. However, these communities are often weakly connected, leading to duplication of effort and missed opportunities for synergy. Strengthening collaboration through shared projects, coordinated funding proposals, and common research agendas will be essential to address the scale and complexity of the problem.

Finally, there is a need to establish clearer pathways for the translation of materials from laboratory research to operational deployment. This includes not only technical validation, but also consideration of issues such as manufacturing scalability, long-term reliability, and integration into existing systems. Developing these pathways will require closer engagement with end-users and facilities, as well as a shift towards application-driven research frameworks that prioritise deployability alongside performance.

Taken together, these needs define a roadmap for the community, emphasising standardisation, data quality, realistic testing, and collaborative development. Addressing them will be critical to unlocking the full potential of low-density materials in future accelerator systems and ensuring that recent advances can be translated into reliable and impactful technologies.

## 3. Production and Treatment Capabilities

Low-density materials for accelerator applications are enabled not only by their intrinsic properties, but by the ability to reliably manufacture and process them with controlled structure across multiple length scales. In recent years, significant progress has been made in the development of production routes for carbon-based systems, particularly carbon nanotube fibres, graphene, and related nanostructured materials. However, as highlighted in the preceding sections, the performance of these materials is strongly dependent on synthesis conditions, post-processing treatments, and the resulting mesoscale architecture. This makes production and treatment capabilities a central factor in determining their suitability for accelerator environments.

### 3.1. Current Processing Routes

**Figure 4:**
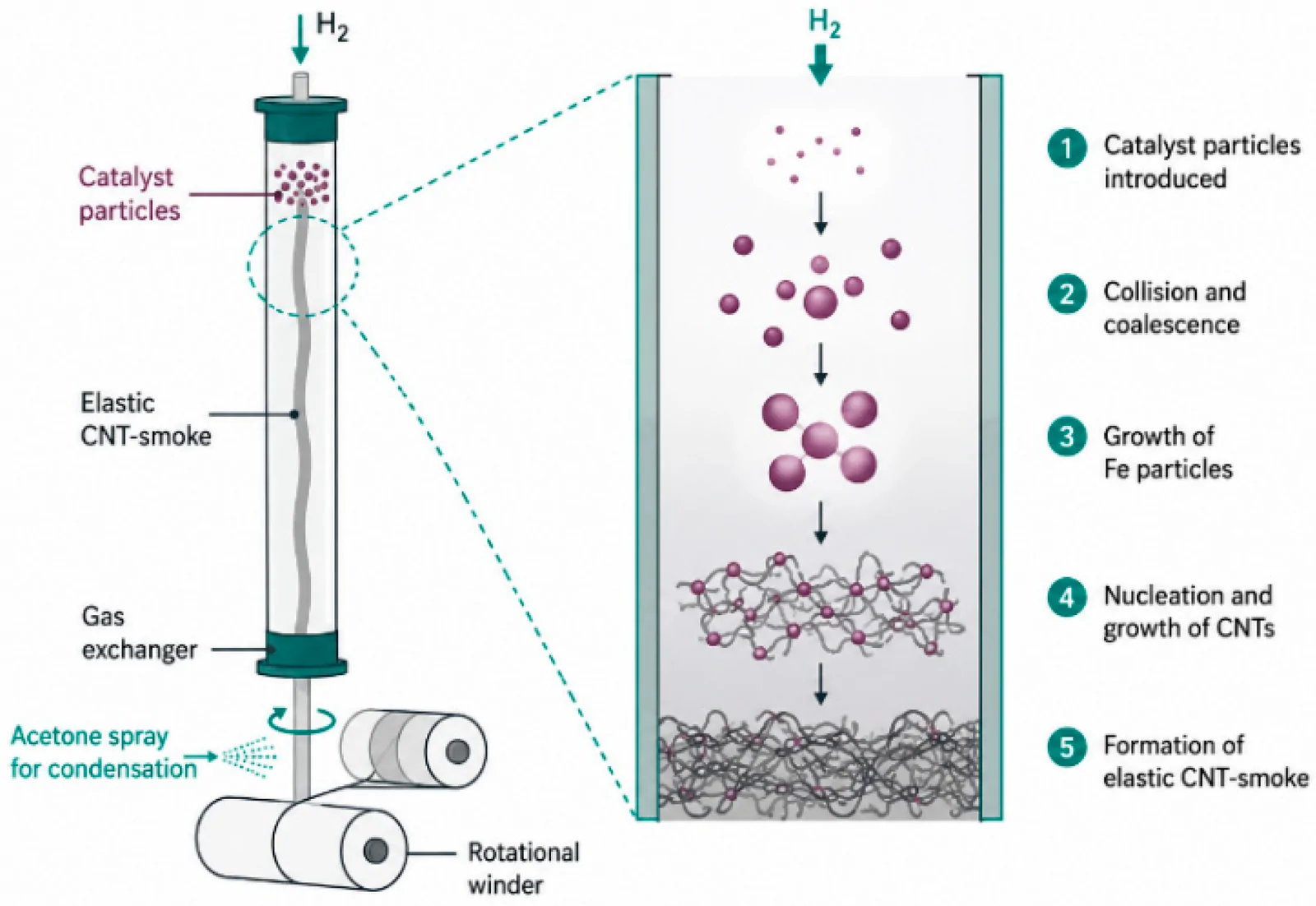
Direct CNT fibre spinning from gas precursor. Adapted from workshop presentation material provided by J.A. Elliott, Low-Density Materials Workshop 2026.

A range of synthesis approaches are currently used to produce low-density materials, with carbon-based systems dominating the field. For CNT-based materials, floating catalyst chemical vapour deposition (FC-CVD) and related gas-phase growth methods are widely employed, enabling the continuous production of fibres, aerogels, and films with relatively low density and high aspect ratio (Figure 4). Variations in precursor chemistry, catalyst composition, and reactor conditions allow some level of control over nanotube structure, including diameter, wall number, and degree of entanglement [3,27]. However, these processes are inherently dynamic, and small fluctuations in temperature, flow conditions, or precursor supply can lead to significant variation in the resulting material.

Alternative routes include liquid-phase processing and spinning techniques, in which CNTs are dispersed and reassembled into fibres or films. These approaches can offer improved control over alignment and packing density, particularly when combined with drawing or stretching processes, but are often limited by defect introduction, solvent effects, and scalability challenges. In both cases, the resulting material properties are determined not only by the nanotubes themselves, but by the quality of inter-tube contacts and the degree of structural organisation achieved during processing [3,28].

For two-dimensional materials such as graphene, synthesis is typically based on chemical vapour deposition (CVD) or exfoliation approaches, followed by transfer and integration into functional structures. While high-quality graphene can be produced at laboratory scale, maintaining uniformity and integrity during transfer and integration remains a significant challenge, particularly for large-area or suspended configurations required in accelerator applications [29].

Post-processing treatments play a critical role across all material systems. Techniques such as thermal annealing, chemical purification, and mechanical densification are commonly used to remove impurities, improve alignment, and enhance interfacial bonding. For CNT fibres, acid treatments and heat-based processing can significantly reduce catalyst residues and amorphous carbon content, while also modifying mechanical and electrical properties. However, these treatments can introduce additional variability if not carefully controlled, and may affect long-term stability or compatibility with accelerator environments [3,30].

### 3.2. Capability Assessment

Current production capabilities demonstrate that low-density materials with highly promising properties can be realised, particularly at laboratory or pilot scale. CNT fibres, for example, can achieve low densities combined with high tensile strength, good electrical conductivity, and excellent thermal resilience, making them strong candidates for applications such as beam diagnostics and intercepting devices. Similarly, graphene and related two-dimensional materials offer extremely low interaction thickness and tuneable functional properties, opening new possibilities for advanced sensing and interaction-based systems.

A key strength of modern processing approaches is the ability to tailor material architecture across hierarchical length scales. Control over alignment, porosity, and bundle structure allows properties to be tuned in ways that are not accessible with conventional materials. Alignment has been shown to be a dominant factor in determining mechanical and transport properties, while packing density and defect distribution strongly influence thermal response and durability.

Despite these advances, the achievable performance remains highly sensitive to process conditions, and the range of properties reported in the literature reflects significant variability rather than well-defined capability limits. As such, while the potential of these materials is clear, their performance envelope is not yet fully controlled or predictable.

### 3.3. Limitations and Bottlenecks

**Figure 5:**
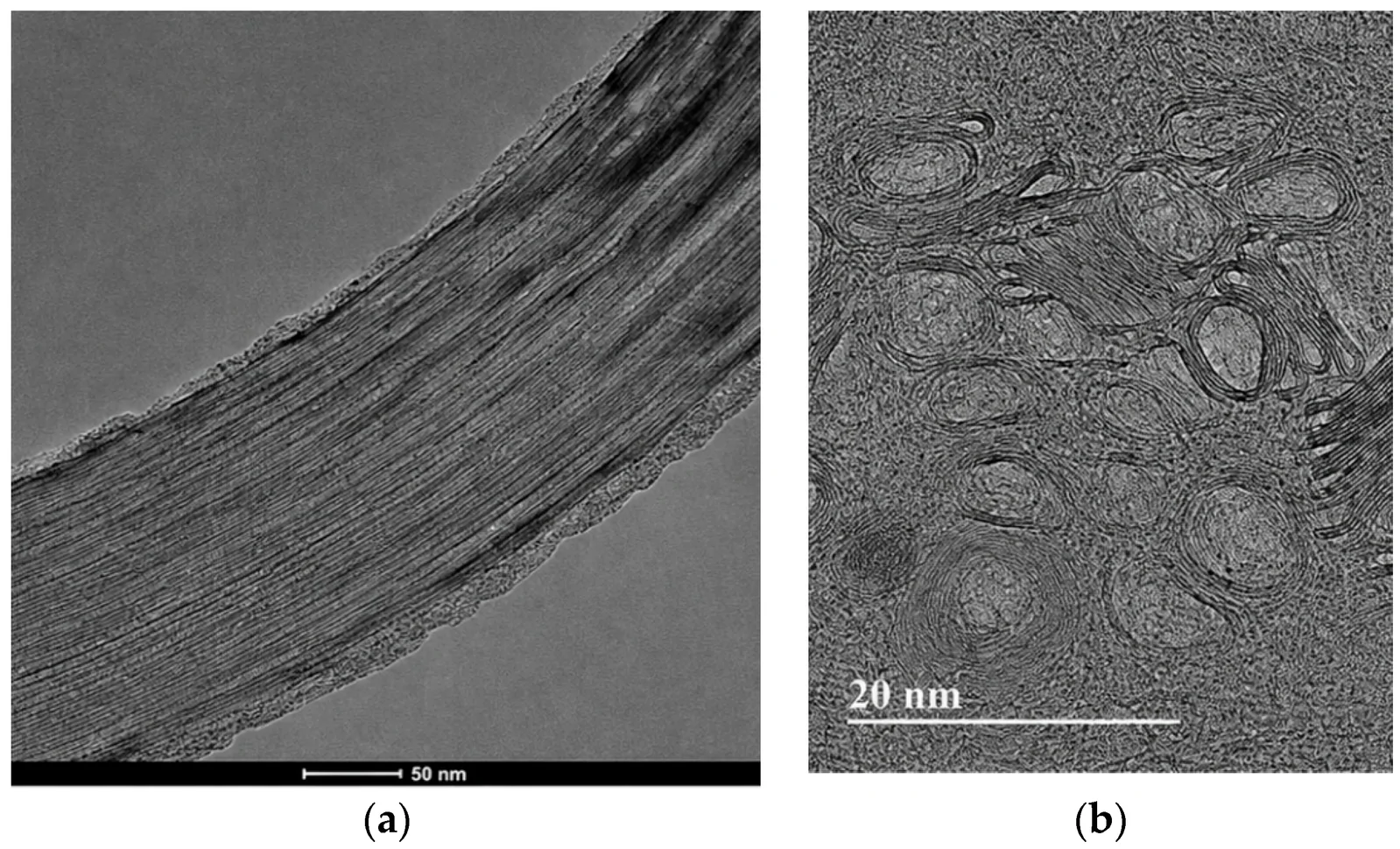
Examples of collapsed CNTs (**a**) Adapted from workshop presentation material provided by A. Mikhalchan at the Low-Density Materials Workshop 2026 (**b**) [31].

The most significant limitation in current production capabilities is the lack of reproducibility (Figure 5). As highlighted in earlier sections, small variations in synthesis parameters can lead to substantial differences in material structure and performance. This is particularly evident in CNT-based systems, where factors such as catalyst stability, precursor decomposition, and flow dynamics introduce variability that is difficult to monitor or control in real time [27,29]. The result is that nominally identical production processes may yield materials with significantly different properties.

Another key bottleneck is the presence of impurities and structural heterogeneity. Residual catalyst particles, amorphous carbon, and incomplete bonding between structural elements can dominate material behaviour, particularly under extreme conditions. While purification and post-processing techniques can mitigate some of these effects, they often introduce trade-offs in terms of damage, scalability, or cost [3,30].

Scaling production to application-relevant volumes presents a further challenge. Many high-performance materials are demonstrated at small scale, where process control is more easily maintained. However, translating these processes to larger-scale or continuous production introduces additional complexity, including variations in reactor behaviour, handling-induced damage, and limitations in quality control. This gap between laboratory-scale demonstration and industrial-scale production remains a major barrier to deployment [24].

Finally, integration with downstream processes represents an additional constraint. Materials must be compatible with machining, mounting, and handling procedures, as well as with the environmental conditions of accelerator systems. Fragility, sensitivity to contamination, and difficulties in forming consistent geometries all limit the practical usability of otherwise promising materials.

## 4. Characterisation and Simulation

Understanding and predicting the behaviour of low-density materials in accelerator environments requires a combination of advanced experimental characterisation and robust modelling approaches. While significant progress has been made in both areas, the workshop discussions highlighted persistent challenges in linking data across length scales, ensuring consistency between measurements, and establishing validated predictive frameworks. These limitations directly impact the ability to design, optimise, and deploy materials under realistic operating conditions.

### 4.1. Experimental Characterisation

Experimental techniques for the characterisation of low-density materials have advanced substantially in recent years, particularly in their ability to probe structure and properties across multiple length scales. For carbon-based systems, a wide range of methods are now routinely employed, including electron microscopy, Raman spectroscopy, X-ray diffraction, and spectroscopic techniques such as X-ray photoelectron spectroscopy and energy dispersive spectroscopy. These approaches provide detailed insight into nanotube structure, bundle organisation, defect density, and the presence of residual impurities, allowing for increasingly sophisticated analysis of structure–property relationships.

**Figure 6:**
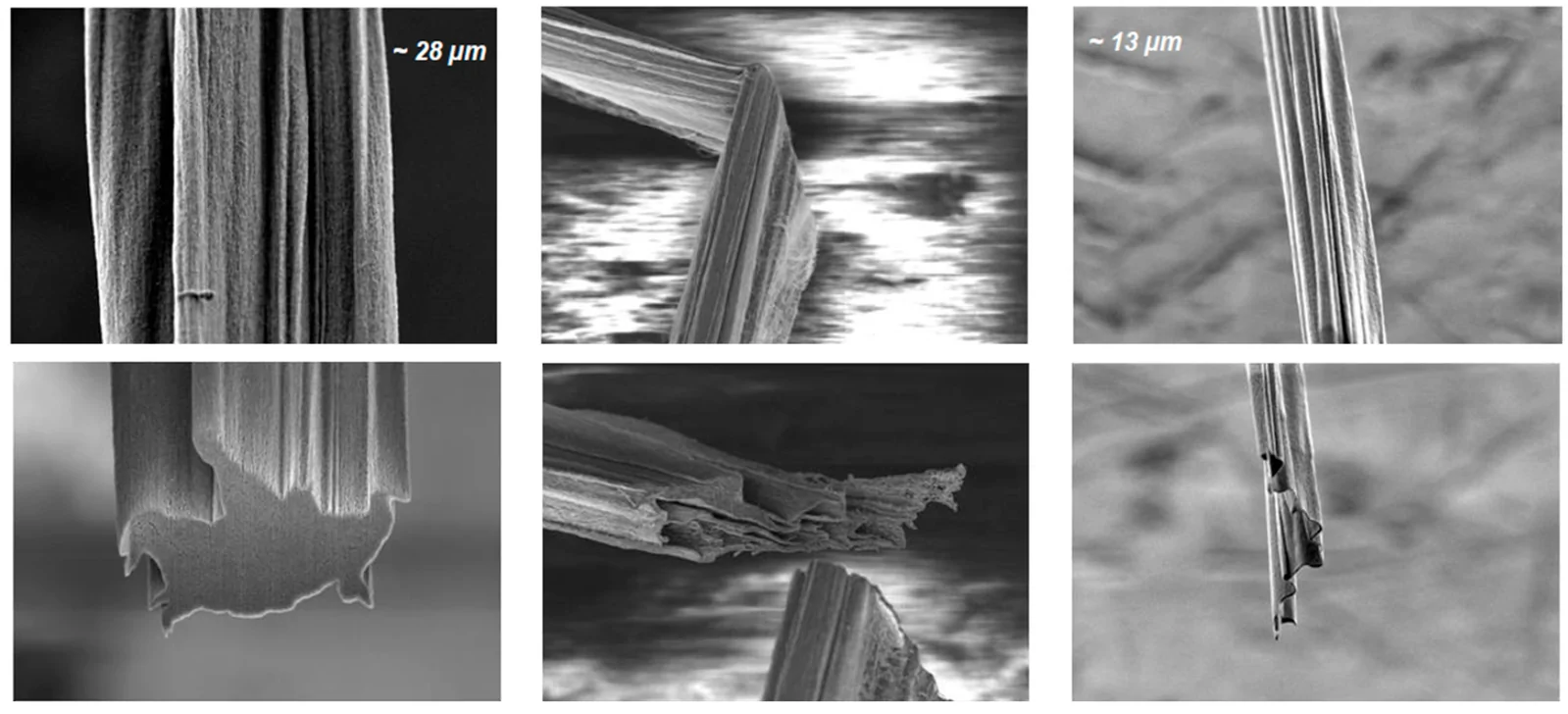
Multiscale characterisation of CNT yarns revealing internal structure and impurities; Images courtesy of Anité Pérez Fontenla (CERN).

At the mesoscale, techniques such as Focused Ion Beam–Scanning Electron Microscopy (FIB–SEM) cross-sectioning and tomography have proven particularly valuable in revealing internal architecture, including packing density, porosity, and heterogeneity (Figure 6). These measurements are critical, as properties in CNT-based materials are often governed not by individual nanotubes, but by their collective arrangement and interconnections. However, interpretation of these datasets remains non-trivial, particularly where structures are highly anisotropic or vary across the sample.

A further important development is the increasing use of in situ and operando techniques, where materials are characterised under mechanical load, thermal cycling, or irradiation. These approaches provide direct insight into deformation mechanisms, damage evolution, and structure–property coupling under conditions closer to those encountered in accelerator systems. Nevertheless, such experiments remain technically challenging and are often limited in throughput and accessibility.

A key limitation across experimental characterisation is the lack of standardisation. Measurements are frequently carried out using different methods, sample preparation protocols, and analysis approaches, making direct comparison between studies difficult. In addition, many techniques probe only a subset of relevant length scales or properties, leading to incomplete datasets that cannot fully describe material behaviour [24,32].

### 4.2. Modelling and Simulation

Modelling plays an increasingly important role in understanding and predicting the behaviour of low-density materials, particularly given the complexity of their structure and the extreme nature of accelerator environments. A wide range of approaches is currently employed, spanning atomistic simulations, mesoscale models, and continuum-level descriptions.

**Figure 7:**
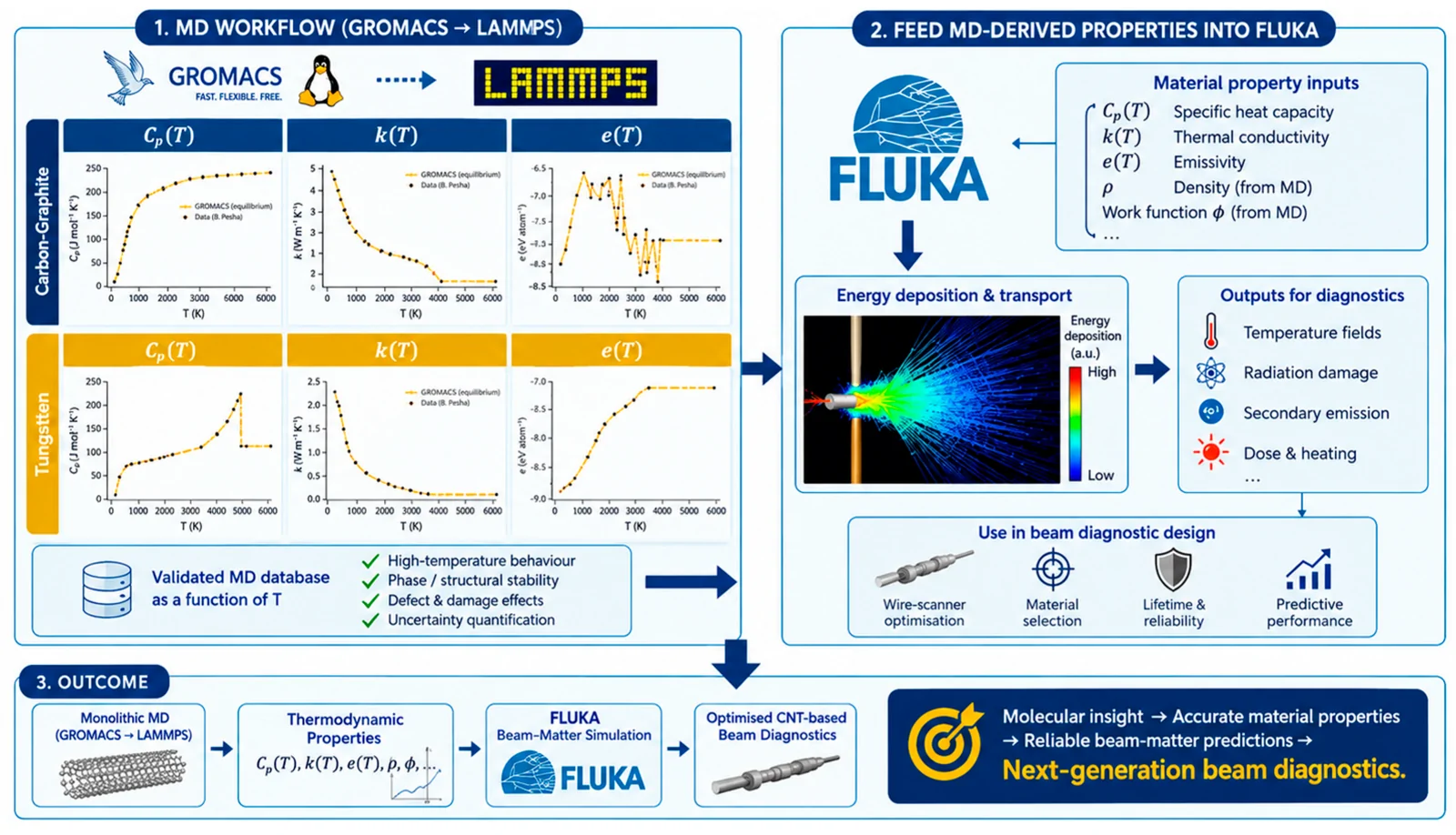
Multiscale modelling approaches linking structure to properties in CNT systems. Image courtesy of Carmelo Herdes Moreno (University of Bath). The graphical layout and certain visual elements were developed with the assistance of Microsoft 365 Copilot and subsequently reviewed and edited by the authors.

At smaller length scales, atomistic and molecular dynamics methods provide insight into fundamental mechanisms such as bonding, defect formation, and local thermal transport (Figure 7). These approaches are particularly useful for understanding the intrinsic properties of nanotubes and graphene, and for exploring the effects of defects and functionalisation. However, their applicability is limited by computational scale, making it difficult to directly translate results to macroscopic systems.

At the mesoscale, modelling efforts focus on network behaviour, including alignment, packing, and inter-tube interactions. These models aim to capture the emergent properties of hierarchical materials, which are often dominated by collective effects rather than individual constituents. However, accurately representing real material structures remains challenging, particularly in the presence of defects and heterogeneity, which are difficult to quantify experimentally.

At the system level, continuum models are used to simulate beam–material interaction, including energy deposition, thermal response, and mechanical behaviour structure [12]. These models are essential for the design of accelerator components, but their accuracy depends strongly on the quality of input data, which is often uncertain or incomplete. This leads to significant variability in predicted performance, particularly for materials that are complex or poorly characterised.

A recurring issue is the difficulty of bridging length scales. While individual modelling approaches can provide insight at specific scales, integrating these into a coherent multiscale framework remains an open challenge. This is particularly important for low-density materials, where behaviour is governed by interactions across scales from the atomic to the macroscopic [8,33].

### 4.3. Data and Validation Challenges

A central theme emerging from the workshop is the gap between available experimental data and the requirements of modelling and design. While large amounts of data are generated, they are often not directly usable as model inputs, either because key parameters are missing, or because measurements are not representative of real operating conditions. This is particularly problematic for properties such as thermal conductivity, emissivity, and damage thresholds, which are highly sensitive to structure and environment.

Another challenge lies in validation. Models are often validated against limited datasets or simplified experiments, which may not capture the complexity of real systems. As a result, predictive capability remains uncertain, particularly when extrapolating to new materials or operating regimes. This limits confidence in simulation-driven design and slows the adoption of new materials in critical applications.

There is also a need for improved data integration and traceability. Linking material properties to synthesis conditions, processing history, and microstructural features is essential for understanding variability and enabling reproducibility. However, such information is rarely captured in a systematic way, making it difficult to build comprehensive datasets or develop data-driven approaches.

Taken together, these challenges highlight the need for a more coordinated and structured approach to characterisation and modelling. This includes the development of standardised measurement protocols, improved integration between experimental and computational efforts, and the creation of shared datasets that capture the complexity of real materials. Addressing these issues will be essential to move from qualitative understanding to predictive capability, enabling the reliable design and deployment of low-density materials in accelerator systems.

## 5. Cross-Cutting Technical Challenges

**Figure 8:**
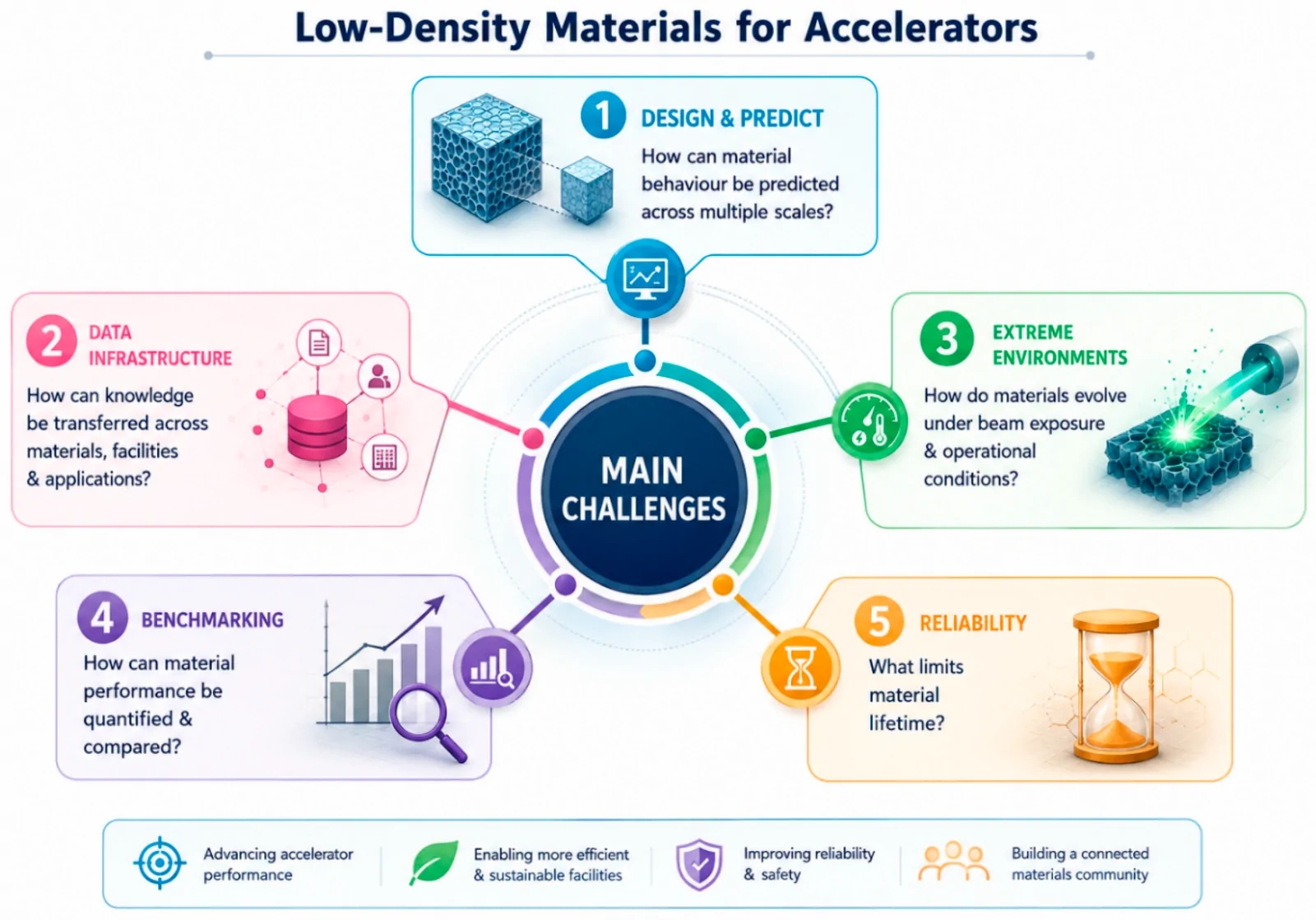
Five key research challenges associated with low-density materials for accelerator applications. The figure was developed from an initial concept provided by the authors and refined with the assistance of Microsoft 365 Copilot.

While the preceding sections have addressed challenges in applications, production, and characterisation separately, several fundamental issues emerge that cut across all stages of development and deployment (see Figure 8). These challenges reflect the intrinsic complexity of low-density materials, where performance is not governed by a single parameter, but by the interaction of structure, processing, and environment across multiple length scales. Understanding and addressing these cross-cutting issues is essential for translating material potential into reliable accelerator technologies.

### 5.1. Multiscale Understanding

A central challenge is the need to establish a coherent understanding of material behaviour across length scales. In low-density materials such as CNT fibres and graphene-based systems, properties originate at the atomic scale but are expressed through mesoscale organisation and ultimately determine macroscopic performance. Features such as alignment, porosity, bundle structure, and defect connectivity play a dominant role, effectively acting as the bridge between intrinsic material properties and observable behaviour.

However, these features are difficult to quantify consistently and are often only partially captured by existing characterisation techniques. As a result, there remains a disconnect between nanoscale understanding and system-level performance. Bridging this gap requires not only improved measurement capability, but also the development of frameworks that explicitly link structure across scales, enabling more reliable prediction and control of material behaviour [8,34].

### 5.2. Materials Reliability and Lifetime

A second major challenge concerns the long-term reliability of low-density materials under realistic operating conditions. Accelerator environments expose materials to extreme and often coupled stresses, including high power density, rapid thermal cycling, and intense radiation fields. Under these conditions, degradation mechanisms such as sublimation, structural rearrangement, defect accumulation, and impurity-driven failure can evolve over time.

Importantly, these processes are rarely governed by intrinsic material limits alone. Instead, they are strongly influenced by microstructural features and imperfections introduced during synthesis and processing. This leads to significant variability in lifetime and failure modes, even between materials with similar nominal properties. As a result, predicting durability and establishing safe operating limits remains a major challenge, requiring better integration of experimental data, modelling, and in situ observation [3,22].

### 5.3. Standardisation and Benchmarking

The lack of standardisation across the community represents a persistent barrier to progress. As highlighted in earlier sections, differences in synthesis routes, sample preparation, measurement techniques, and reporting practices make it difficult to compare results between studies or to establish reliable performance benchmarks. This issue is particularly pronounced for low-density materials, where properties are highly sensitive to subtle variations in structure and processing.

Without common standards, it is challenging to distinguish intrinsic material behaviour from artefacts of preparation or measurement. This limits the ability to build shared datasets, validate models, and define meaningful performance targets. Developing standardised protocols for production, characterisation, and data reporting is therefore a critical step towards improving reproducibility and enabling systematic progress across the field [24,32].

### 5.4. Integration into Real Systems

A further cross-cutting challenge lies in the integration of low-density materials into operational accelerator systems. While many materials show promising properties in isolation, their performance can change significantly when incorporated into real devices. Factors such as mounting constraints, environmental exposure, thermal coupling, and interaction with surrounding components all influence behaviour at the system level.

In addition, practical considerations such as handling, robustness, and compatibility with existing infrastructure impose constraints that are not captured in laboratory studies. For example, materials that perform well in controlled experiments may prove difficult to implement due to fragility, variability, or incompatibility with standard fabrication techniques. This highlights the importance of considering system-level requirements early in the development process, and of validating materials under realistic conditions wherever possible [14].

### 5.5. Data, Digitalisation, and AI

An emerging cross-cutting theme is the role of data and digital approaches in accelerating materials development. The complexity and variability of low-density materials make them well suited to data-driven analysis, where large datasets can be used to identify correlations between processing conditions, structure, and performance. Digital twins and machine learning approaches offer the potential to integrate experimental and modelling efforts, enabling more efficient exploration of parameter space and improved predictive capability [35].

However, the effectiveness of these approaches depends critically on the availability of high-quality, standardised data. At present, data fragmentation, inconsistency, and limited traceability represent significant barriers to their adoption. Addressing these issues will require coordinated efforts to develop shared data frameworks and to capture the full context of material production and testing, including uncertainties and variability [26].

Taken together, these cross-cutting challenges highlight that the development of low-density materials is fundamentally a systems problem, requiring coordinated progress across multiple domains. Addressing them will involve not only advances in individual areas such as synthesis or modelling, but also improved integration, standardisation, and collaboration across the community. This provides a natural basis for the emerging opportunities and future directions discussed in the following section.

### 5.6. Emerging Opportunities

The challenges identified across materials development, characterisation, and system integration also define a set of emerging opportunities, where recent advances open new directions for research and application. These opportunities are not confined to incremental improvements in existing systems but instead reflect a broader shift towards materials that are engineered across multiple length scales and actively integrated into accelerator functionality.

At a fundamental level, the increasing ability to control structure across hierarchical scales enables the design of materials with tailored combinations of properties that are not accessible through conventional approaches. In carbon-based systems, this includes control over alignment, porosity, defect content, and functionalisation, allowing simultaneous optimisation of thermal, mechanical, and electrical behaviour. Such capabilities directly address many of the limitations identified in earlier sections, particularly where performance is governed by mesoscale organisation rather than intrinsic material properties [8]. These developments give rise to several key opportunity areas:

1. Advanced beam diagnostics

The continued refinement of low-density materials, particularly CNT fibres and nanostructured thin films, enables the development of highly sensitive, minimally invasive diagnostic systems. Improvements in spatial resolution, reduced beam perturbation, and enhanced durability are expected to extend the operational range of existing techniques such as wire scanners, while also enabling new diagnostic modalities.

2. High-performance intercepting devices

Materials with improved thermal resilience and reduced energy deposition provide opportunities for more robust beam-intercepting components, including screens, stripping foils, and collimation systems. The ability to tailor thermal transport and damage tolerance at the structural level is particularly relevant for next-generation high-intensity machines.

3. Functional and active materials

A significant shift is the transition from passive materials to systems that actively participate in beam interaction. Examples include CNT-based electron emitters, graphene-based transmission structures, and engineered nanostructures for photon or particle interaction. These systems blur the boundary between material and device, enabling new operational concepts [19,36].

4. Structured interaction media and novel accelerator concepts

The use of nanostructured materials, such as CNT forests and graphene-based architectures, introduces the possibility of highly engineered interaction environments. These materials are being explored for applications ranging from compact acceleration platforms to tailored beam–matter interaction regimes, representing a step change in how materials are used within accelerator systems.

5. Data-driven materials development

The increasing availability of experimental and modelling data, combined with advances in digital tools and machine learning, creates opportunities for accelerated materials discovery and optimisation. By linking processing conditions to structure and performance, these approaches have the potential to reduce development time and improve reproducibility, provided that challenges in data quality and standardisation are addressed [24,32].

A common feature across these opportunities is the central role of structure as a design parameter, rather than a by-product of processing. This represents a shift in paradigm, where material performance is engineered through controlled architecture across multiple scales, supported by integrated experimental and modelling approaches. At the same time, realising these opportunities will require progress in the cross-cutting areas identified previously, particularly in reproducibility, standardisation, and data integration. Without such advances, the variability and uncertainty inherent in current material systems will continue to limit their practical impact.

Taken together, these directions highlight the potential for low-density materials not only to enhance existing accelerator technologies, but also to enable new concepts and operational regimes. This provides a clear foundation for defining priorities and strategic actions in the following roadmap section.

## 6. Recommendations and Roadmap

The outcomes of the workshop highlight both the significant progress made in the development of low-density materials and the substantial challenges that remain in translating these advances into reliable accelerator technologies. Addressing these challenges requires a coordinated and strategic approach, combining advances in materials science, experimental validation, modelling, and system-level integration. This section outlines a set of priority actions, structured across short-, medium-, and long-term timescales, to guide future research and collaboration within the community.

### 6.1. Short-Term Priorities

In the near term, the focus should be on addressing the most immediate barriers to reproducibility, comparability, and validation. A key priority is the development of standardised protocols for material synthesis, handling, and characterisation. Establishing agreed procedures for sample preparation, measurement techniques, and data reporting will enable more reliable comparison between studies and support the creation of shared datasets.

In parallel, efforts should be directed towards improving the quality and relevance of material data. This includes systematic measurement of properties under well-defined conditions, with clear traceability to processing history and microstructural features. Emphasis should be placed on properties directly relevant to accelerator performance, such as thermal transport, damage thresholds, and radiation response.

Another important short-term goal is the expansion of testing under realistic or near-operational conditions. Where possible, materials should be evaluated using beam-based experiments, or through representative multi-physics testing that captures the combined effects of thermal, mechanical, and radiation loading. These activities are essential for building confidence in material behaviour and for identifying failure mechanisms at an early stage.

Finally, strengthening communication and coordination across the community represents a low-cost but high-impact action. This includes the establishment of shared platforms for data exchange, collaborative benchmarking exercises, and regular interaction between material developers and end-users.

### 6.2. Medium-Term Goals

Over the medium term, the emphasis should shift towards integration and predictive capability. A major objective is the development of consistent frameworks linking material structure, processing, and performance across multiple length scales. This requires closer coupling between experimental characterisation and modelling, ensuring that data generated at different scales can be effectively combined and used to inform design.

Advances in modelling should focus on improving multiscale integration, enabling the transition from qualitative understanding to quantitative prediction. This includes the development of models that explicitly incorporate mesoscale features such as alignment, porosity, and defect distribution, as well as their evolution under operational conditions.

Scaling production processes represents another critical medium-term goal. Efforts should be directed towards improving process stability, monitoring, and control, particularly for systems such as CNT fibre production where variability remains a major limitation. This may involve the introduction of in situ diagnostics, feedback control systems, and more robust process design methodologies. In addition, the development of application-specific material systems should be prioritised. Rather than pursuing generic performance improvements, materials should be designed and optimised for specific accelerator use cases, considering system-level constraints and operational requirements from the outset.

### 6.3. Long-Term Vision

In the longer term, the goal is to establish low-density materials as reliable, fully integrated components within accelerator systems, supported by predictive design tools and scalable manufacturing processes. Achieving this will require a shift from exploratory research to mature, application-driven development.

A key aspect of this vision is the creation of digital frameworks that integrate materials data, modelling, and experimental validation. Digital twins of material systems and components could enable rapid optimisation, improved reliability, and more efficient translation from design to deployment. The integration of artificial intelligence and machine learning into these frameworks offers further potential to accelerate discovery and reduce uncertainty.

Another long-term objective is the development of next-generation materials and architectures that enable new accelerator concepts. This includes engineered nanostructures, functional materials, and structured interaction media capable of actively shaping beam behaviour. Such developments may lead to fundamentally new approaches to beam diagnostics, acceleration, and particle interaction.

Finally, sustained collaboration and investment will be essential. The multidisciplinary nature of the challenges requires coordinated programmes that bring together expertise from across materials science, accelerator physics, and engineering, supported by dedicated funding and shared infrastructure.

### 6.4. Community Actions

The successful implementation of this roadmap depends on collective action across the community. Several practical steps can be identified:

- Establish collaborative networks focused on low-density materials for accelerator applications, including shared research programmes and coordinated funding proposals.
- Develop and maintain open-access databases capturing material properties, processing conditions, and experimental results.
- Organise benchmarking studies and round-robin experiments to assess reproducibility and validate measurement techniques.
- Promote closer interaction between experimentalists, modellers, and system designers to ensure alignment of objectives and priorities.
- Support training and knowledge exchange to build expertise at the interface between materials science and accelerator technology.

Taken together, these recommendations outline a clear pathway from current capabilities towards the reliable deployment of low-density materials in accelerator systems. By addressing immediate challenges in reproducibility and validation, while investing in longer-term integration and innovation, the community is well positioned to realise the full potential of these materials. The continued alignment of research efforts, infrastructure, and strategic priorities will be essential in achieving this goal.

## 7. Conclusions

This workshop has highlighted the growing importance of low-density materials as enabling technologies for current and next-generation particle accelerator systems. Across applications ranging from beam diagnostics to intercepting devices and emerging functional components, these materials offer clear advantages in reducing beam interaction while maintaining, and in many cases enhancing, thermal, mechanical, and functional performance. At the same time, their development introduces a new level of complexity, where behaviour is governed not only by intrinsic material properties, but by structure and organisation across multiple length scales.

A central finding is that the performance of low-density materials is fundamentally controlled by mesoscale architecture, including alignment, porosity, defect distribution, and interfacial interactions. This represents a shift away from conventional materials design, where composition alone is often the primary consideration. As a result, reproducibility, processing control, and structural characterisation emerge as critical factors, directly influencing material behaviour under the extreme conditions encountered in accelerator environments.

Despite substantial progress in synthesis, characterisation, and modelling, several key barriers remain. Variability in material production, the presence of impurities and structural heterogeneity, and the limited availability of reliable, transferable material data restrict both understanding and deployment. In parallel, the gap between laboratory-scale demonstration and system-level implementation continues to pose significant challenges, particularly in applications where long-term reliability and operational stability are essential.

The discussions further emphasised that these challenges are inherently interconnected. Advances in one area, such as materials processing or experimental characterisation, must be accompanied by corresponding progress in modelling, validation, and system integration. This underscores the need for a coordinated and multidisciplinary approach, bringing together expertise across materials science, accelerator physics, chemistry, and engineering.

At the same time, the opportunities are substantial. Emerging capabilities in hierarchical materials design, nanostructured systems, and data-driven approaches offer new pathways for both improving existing technologies and enabling entirely new concepts. In particular, the transition from passive to functional and active materials represents a significant shift in how materials can contribute to accelerator performance.

Realising this potential will require sustained effort in several key areas, including the development of standardised methodologies, the generation of high-quality and traceable datasets, and the establishment of shared frameworks linking structure, processing, and performance. Equally important is the need to strengthen collaboration across the community, ensuring alignment between material development and application requirements.

In conclusion, low-density materials represent a promising but still maturing field within accelerator science. Their successful integration into future systems will depend not only on continued advances in materials performance, but on the ability to manage complexity, reduce variability, and build confidence through reproducible and validated approaches. The outcomes of this workshop provide a clear foundation for these efforts, outlining both the challenges to be addressed and the opportunities to be realised in the coming years.

## Data Availability

No new data were created or analyzed in this study. Data sharing is not applicable to this article.

## Supplementary Materials

The full workshop programme and supporting materials are available at https://indico.cern.ch/event/1660653/ (accessed on 15 September 2026).

## Abbreviations

The following abbreviations are used in this manuscript:

CERN	European Organization for Nuclear Research
CNT	Carbon Nanotube
FC-CVD	Floating Catalyst Chemical Vapour Deposition
FCC	Future Circular Collider
FCC-hh	Future Circular Collider Hadron-Hadron
FEL	Free-Electron Laser
FIB-SEM	Focused Ion Beam Scanning Electron Microscopy
HL-LHC	High-Luminosity Large Hadron Collider
ISOLDE	Isotope Separator On-Line Device
LHC	Large Hadron Collider

## Appendix A

### Appendix A.1. Workshop Overview

The workshop was organised over two days and structured around a series of thematic sessions designed to provide a coherent progression from application requirements through to enabling technologies and future directions. The programme began with an introductory session, followed by Session 1, which focused on applications, the state of the art, and the needs of the community across accelerator systems. This was followed by Session 2, addressing current and emerging capabilities in the production and treatment of low-density materials, including advances in carbon-based systems and scalable manufacturing approaches. On the second day, Session 3 explored simulation and characterisation, highlighting developments in experimental techniques, multiscale modelling, and data-driven approaches. The workshop concluded with Session 4, a wrap-up session dedicated to funding opportunities, strategic alignment, and defining future collaborative actions. The programme also included informal discussions and networking opportunities to support cross-disciplinary exchange.

### Appendix A.2. Workshop Program

**Table A1:** Overview of workshop sessions and talks.

Speaker(s)	Presentation Title	Chair
INTRODUCTION AND WELCOME TO BATH		
Raymond Veness & Alexander J. G. Lunt	Introduction and Welcome	—
SESSION 1—Applications, State of the Art and Needs in the Community		
Francesca M. Addesa	Minimally Invasive Nano-Fabricated Wire Scanner for FEL Operations	Chiara Pasquino
Alexander J. G. Lunt	Wire Scanners in Hadron Machines	
Federico Roncarolo	Beam Imaging and Low-Density Materials for Screens under Extreme Beam Conditions	
Simon Stegemann	Nanostructured and Low-Dimensional Materials for Accelerator Targets	Thierry Stora
Wim Weterings	Applications of Low-Density Materials for Beam Stripping in High-Intensity Accelerators	
Emi Yamakawa	Performance and Operation of Carbon Nanotube Materials at ISIS	
SESSION 2—Current and Potential Capabilities for Production and Treatment		
Matteo Pasquali	Carbon Nanotube Fibre Production and Scale-Up	Philippe Poulin
James A. Elliott	CNT-Based Materials: Production, Synthesis and Structure–Property Relationships	
Yoku Inoue	Production and Processing of Ultra-Long Carbon Nanotubes	
Anastasiia Mikhalchan	CNT Macromaterials: Challenges in Production, Testing and Properties	Alexander J. G. Lunt
Shiqi Huang	Å-Scale Pore Generation in Functionalised Suspended Graphene	
Habib Rostami	Nonlinear Photocurrent Generation in Two-Dimensional Materials	
SESSION 3—Simulation and Characterisation		
Fernando Lomello	Accelerated Materials Design through Digital Twins and Artificial Intelligence	Stefano Sgobba
Anité Pérez Fontenla	Advanced Microscopy and Characterisation Techniques at CERN	
Marcel Himmerlich	Characterisation of Low-Dimensional Carbon Materials Using Low-Energy Electrons	
Carmelo Herdes Moreno	Multiscale Modelling of Carbon Nanotube Systems for Accelerator Applications	Mariusz Sapinski
Ryan Paul Camilleri	Thermal Response of Beam Diagnostic Wires under High-Power Hadron Beams	
Emmanuel Flahaut	Safety of Engineered Carbon Nanomaterials: Environmental and Health Considerations	
SESSION 4—Wrap-Up, Funding Strategy and Next Steps		
Bifeng Lei & Carsten P. Welsch	Carbon Nanotube Forests as a Platform for Ultra-Compact High-Energy Accelerators	Raymond Veness
Thierry Stora	European Funding Opportunities and Strategic Pathways	
Philippe Poulin	State of the Art and Future Directions in Low-Density Materials	
Raymond Veness	Workshop Wrap-Up and Actions	

### Appendix A.3. Participants

**Table A2:** List of Conference Participants.

Name	Affiliation
Ana Teresa Perez Fontenla	CERN
Benjamin Moser	CERN
Chiara Pasquino	CERN
Federico Roncarolo	CERN
Marcel Himmerlich	CERN
Raymond Veness	CERN
Simon Thomas Stegemann	CERN
Stefano Sgobba	CERN
Thierry Stora	CERN
Wim Weterings	CERN
Elisabeth-Sena Welker	CERN/University of Bath
Adriana Rossi	CERN
Adelina Ilie	University of Bath
Alexander J. G. Lunt	University of Bath
Antonio Pellegrino	University of Bath
Carmelo Herdes	University of Bath
Habib Rostami	University of Bath
Shiqi Huang	University of Bath
Adam Wild	University of Bath
Daniel Wolverson	University of Bath
Francesca Maria Addesa	Paul Scherrer Institute (PSI)
Mariusz Sapinski	Paul Scherrer Institute (PSI)
Ryan Paul Camilleri	CERN/University of Malta
James A. Elliott	University of Cambridge
Emmanuel Flahaut	CNRS/University of Toulouse
Fernando Lomello	CEA DRF/IRFU
Anastasiia Mikhalchan	IMDEA Materials Institute
Matteo Pasquali	Rice University/Carbon Hub
Bifeng Lei	University of Liverpool/Cockcroft Institute
Carsten Peter Welsch	University of Liverpool/Cockcroft Institute
Yoku Inoue	Shizuoka University
Hisashi Sugime	Kindai University
Emi Yamakawa	STFC/ISIS Neutron and Muon Source

## References

[1] Apollinari G., Béjar Alonso I., Brüning O., Fessia P., Lamont M., Rossi L., Tavian L. (2017). High-Luminosity Large Hadron Collider (HL-LHC).

[2] Abada A., Abbrescia M., AbdusSalam S.S., Abdyukhanov I., Abelleira Fernandez J., Abramov A., Aburaia M., Acar A.O., Adzic P.R., Agrawal P. (2019). FCC-Hh: The Hadron Collider. Eur. Phys. J. Spec. Top..

[3] Aliana-Cervera G., Mattia D., Pasquino C., Veness R., Lunt A.J.G. (2025). Carbon Nanotube Wires for Accelerator Applications. Mater. Des..

[4] Lu W., Zu M., Byun J.-H., Kim B.-S., Chou T.-W. (2012). State of the Art of Carbon Nanotube Fibers: Opportunities and Challenges. Adv. Mater..

[5] Krasheninnikov A.V., Banhart F. (2007). Engineering of Nanostructured Carbon Materials with Electron or Ion Beams. Nat. Mater..

[6] Kateris N., Kloza P., Qiao R., Elliott J.A., Boies A.M. (2020). From Collisions to Bundles: An Adaptive Coarse-Grained Model for the Aggregation of High-Aspect-Ratio Carbon Nanotubes. J. Phys. Chem. C.

[7] Xiong Z., Zhong L., Wang H., Li X. (2021). Structural Defects, Mechanical Behaviors, and Properties of Two-Dimensional Materials. Materials.

[8] Wang L., Tricard N., Chen Z., Deng S. (2025). Progress in Computational Methods and Mechanistic Insights on the Growth of Carbon Nanotubes. Nanoscale.

[9] Elliott J.A., Sandler J.K.W., Windle A.H., Young R.J., Shaffer M.S.P. (2004). Collapse of Single-Wall Carbon Nanotubes Is Diameter Dependent. Phys. Rev. Lett..

[10] Koziol K., Vilatela J., Moisala A., Motta M., Cunniff P., Sennett M., Windle A. (2007). High-Performance Carbon Nanotube Fiber. Science.

[11] Prabhakar P., Gopi S., Thomas S., Gopi S., Aryamol K.S., Thomas S., Haponiuk J.T., Maria H.J. (2025). Chapter 12—Simulation and Multiscale Modeling of Carbon Nanomaterials. Nanostructured Carbon Materials from Plant Extracts.

[12] Mariet A., Perez Fontenla A.T., Gabrion X., Salomon C., Veness R., Devel M. (2022). Mechanical Characterization of Yarns Made from Carbon Nanotubes for the Instrumentation of Particle Beams at CERN. Nucl. Instrum. Methods Phys. Res. Sect. Accel. Spectrometers Detect. Assoc. Equip..

[13] Bigland H., Huber J.E., Veness R., Cocks A.C.F. (2020). Wire Materials for Scanners in the Large Hadron Collider: An Unusual Materials Selection Problem. Adv. Eng. Mater..

[14] Veness R., Burger S., Aliana-Cervera G., Pasquino C. Development of Low Density Materials for Beam Intercepting Instruments. Proceedings of the International Beam Instrumentation Conference (IBIC 2025).

[15] Weterings W., Bracco C., Jorat L., Renner E. (2023). Installation and Commissioning of the Stripping Foil System in the New CERN PS Booster 160 MeV H^−^ Injection Region. EPJ Web Conf..

[16] Ramos J.P., Gottberg A., Augusto R.S., Mendonca T.M., Riisager K., Seiffert C., Bowen P., Senos A.M.R., Stora T. (2016). Target Nanomaterials at CERN-ISOLDE: Synthesis and Release Data. Nucl. Instrum. Methods Phys. Res. Sect. B Beam Interact. Mater. At..

[17] Hassan J.Z., Raza A., Babar Z.U.D., Qumar U., Kaner N.T., Cassinese A. (2023). 2D Material-Based Sensing Devices: An Update. J. Mater. Chem. A.

[18] Politano G.G., Parthenios J. (2026). Recent Advances in Graphene and Other Two-Dimensional Materials. Crystals.

[19] Resta-López J., Apsimon O., Bonatto A., Bonțoiu C., Galante B., Welsch C., Xia G. (2022). Application of Nanostructures and Metamaterials in Accelerator Physics.

[20] Posos T.Y., Cook J., Baryshev S.V. (2024). Bright Spatially Coherent Beam from Carbon-Nanotube Fiber Field-Emission Cathode. Phys. Rev. Appl..

[21] Krasheninnikov A.V., Batzill M., Delenda A.-A., Drndić M., Ewels C., Franke K.J., Ghorbani-Asl M., Holleitner A., Jorio A., Kaiser U. (2026). Defects and Defect-Mediated Engineering of Two-Dimensional Materials: Challenges and Open Questions. Beilstein J. Nanotechnol..

[22] Mariet A. (2023). Study of the Effects of Copper-Coating and Proton Irradiation at 440 GeV on the Mechanical Properties of Carbon Nanotube Wires for Particle Beam Instrumentation at CERN Etude Des Effets Du Cuivrage et de l’Irradiation Par Proton à 440 GeV sur les Propriétés Mécaniques de Fils en Nanotubes de Carbone pour l’instrumentation des Faisceaux de Particules Au CERN. Ph.D. Thesis.

[23] Kaselouris E. (2025). Advances in Modelling and Simulation of Materials in Applied Sciences. Materials.

[24] Bøggild P., Booth T.J., Jessen B.S., Shivayogimath A., Lassaline N., Hofmann S., Burton O., Daasbjerg K., Smith A., Nørgaard K. (2025). Protocols and Tools to Enable Reproducibility in 2D Materials Research. Nat. Rev. Phys..

[25] Rajan K. (2005). Materials Informatics. Mater. Today.

[26] Sivan D., Satheesh Kumar K., Abdullah A., Raj V., Misnon I.I., Ramakrishna S., Jose R. (2024). Advances in Materials Informatics: A Review. J. Mater. Sci..

[27] Hou P., Zhang F., Zhang L., Liu C., Cheng H. (2022). Synthesis of Carbon Nanotubes by Floating Catalyst Chemical Vapor Deposition and Their Applications. Adv. Funct. Mater..

[28] Yang Z., Yang Y., Huang Y., Shao Y., Hao H., Yao S., Xi Q., Guo Y., Tong L., Jian M. (2024). Wet-Spinning of Carbon Nanotube Fibers: Dispersion, Processing and Properties. Natl. Sci. Rev..

[29] Dhiman G., Kumar S., Kumar R., Brajpuriya R. (2024). An Improved CVD Design for Graphene Growth and Transfer Improvements. J. Electron. Mater..

[30] Madikere Raghunatha Reddy A.K., Darwiche A., Reddy M.V., Zaghib K. (2025). Review on Advancements in Carbon Nanotubes: Synthesis, Purification, and Multifaceted Applications. Batteries.

[31] Vila M., Hong S., Park S., Mikhalchan A., Ku B.-C., Hwang J.Y., Vilatela J.J. (2021). Identification of Collapsed Carbon Nanotubes in High-Strength Fibers Spun from Compositionally Polydisperse Aerogels. ACS Appl. Nano Mater..

[32] Celano U., Schmidt D., Beitia C., Orji G., Davydov A.V., Obeng Y. (2024). Metrology for 2D Materials: A Perspective Review from the International Roadmap for Devices and Systems. Nanoscale Adv..

[33] Fish J., Wagner G.J., Keten S. (2021). Mesoscopic and Multiscale Modelling in Materials. Nat. Mater..

[34] Karakasidis T.E., Charitidis C.A. (2007). Multiscale Modeling in Nanomaterials Science. Mater. Sci. Eng. C.

[35] Kalidindi S.R., Buzzy M., Boyce B.L., Dingreville R. (2022). Digital Twins for Materials. Front. Mater..

[36] Tranquille G., Cenede J., Galante B., Welker E.-S. (2024). Development of a Field Emission Electron Gun for Low Energy Electron Cooling.

